# Farmed Mussels: A Nutritive Protein Source, Rich in Omega-3 Fatty Acids, with a Low Environmental Footprint

**DOI:** 10.3390/nu13041124

**Published:** 2021-03-29

**Authors:** Elham Yaghubi, Stefano Carboni, Rhiannon M. J. Snipe, Christopher S. Shaw, Jackson J. Fyfe, Craig M. Smith, Gunveen Kaur, Sze-Yen Tan, David. Lee Hamilton

**Affiliations:** 1 Department of Cellular and Molecular Nutrition, School of Nutritional Sciences and Dietetics, Tehran University of Medical Sciences (TUMS), Tehran, Iran; elhamyaghoobi92@gmail.com; 2 Institute of Aquaculture, Faculty of Natural Sciences, University of Stirling, Pathfoot Building, Stirling FK9 4LA, UK; stefano.carboni@stir.ac.uk; 3 Centre for Sport Research, Deakin University, Geelong 3216, Australia; r.snipe@deakin.edu.au; 4 Institute for Physical Activity and Nutrition (IPAN), School of Exercise and Nutrition Sciences, Deakin University, Geelong 3216, Australia; chris.shaw@deakin.edu.au (C.S.S.); jackson.fyfe@deakin.edu.au (J.J.F.); gunveen.kaur@deakin.edu.au (G.K.); szeyen.tan@deakin.edu.au (S.-Y.T.); 5 Institute for Mental and Physical Health and Clinical Translation (IMPACT), School of Medicine, Deakin University, Geelong 3216, Australia; craig.smith@deakin.edu.au

**Keywords:** mussels, omega-3 fatty acids, omega-3 index, food first, nutrition, n-3

## Abstract

The world’s ever-growing population presents a major challenge in providing sustainable food options and in reducing pressures on the Earth’s agricultural land and freshwater resources. Current estimates suggest that agriculture contributes ~30% of global greenhouse gas (GHG) emissions. Additionally, there is an increased demand for animal protein, the production of which is particularly polluting. Therefore, the climate-disrupting potential of feeding the planet is likely to substantially worsen in the future. Due to the nutritional value of animal-based protein, it is not a simple solution to recommend a wholesale reduction in production/consumption of animal proteins. Rather, employing strategies which result in the production of low carbon animal protein may be part of the solution to reduce the GHGs associated with our diets without compromising diet quality. We suggest that farmed mussels may present a partial solution to this dilemma. Mussel production has a relatively low GHG production and does not put undue pressure on land or fresh water supplies. By drawing comparisons to other protein sources using the Australian Food and Nutrient Database and other published data, we demonstrate that they are a sustainable source of high-quality protein, long-chain omega-3 fatty acids, phytosterols, and other key micronutrients such as B-12 and iron. The aim of this review is to summarise the current knowledge on the health benefits and potential risks of increasing the consumption of farmed mussels.

## 1. Introduction

Agriculture is estimated to contribute approximately 30% of the total greenhouse gas (GHG) emissions [1]. The production of animal products (milk, meat and eggs) constitutes a large component (56–58% [2]) of global agricultural derived GHGs [3] and modelling experiments suggest that shifting our food choices away from animal sources could have a significant impact on GHG emissions in addition to conserving land and water [4,5]. For instance, if the average diet in the UK was shifted to a vegan diet, it would lead to a reduction in GHG emissions of 2.4 kg CO_2e_ person^−1^ day^−1^ (a 28% reduction in diet related GHG emissions), which is equivalent to 50% of the UK’s passenger cars’ exhaust emissions in 2012 [5]. Therefore, dietary changes could have substantial impacts on global climate change targets and the sustainability of our food systems if they were to be implemented widely. Consuming more plant-based foods in the habitual diet is echoed in the EAT-Lancet Commission exploring the development of healthy diets from food systems that are sustainable in the long term [6]. However, whilst vegan and plant-based diets have been associated with a reduced risk of ischemic heart disease and cancer [7], there are significant concerns around the nutrient adequacy (iron, B12 and protein in particular) of the vegan diet [8]. There is recent evidence that long term vegans have an increased risk of bone fractures [9], suggesting that the long-term removal of animal products from the diet may negatively impact bone health. There is also significant concern that a non-supplemented vegan diet cannot support the nutritional needs of pregnant women, children, or the elderly [10,11]. This presents a major dilemma whereby improving our planet’s climate and ecological health requires a decreased reliance on meat, fish poultry and dairy, yet for the optimal health of our population we likely need to maintain at least a minimal intake of animal products.

Vegetarianism/veganism are not the only dietary strategies suggested to be more environmentally friendly than the typical omnivorous diet. An alternative strategy is to replace terrestrial meat and poultry with seafood. A lacto-ovo-pescatarian diet, whilst not as low on GHG emissions as some plant-based diets, is substantially lower for GHG emissions than a standard omnivorous diet [5] and could represent a transition strategy towards a more plant-based diet for some. Furthermore, multiple health agencies encourage the increased consumption of fish to improve the overall healthfulness of our diet. Unfortunately, a pescatarian diet is not without environmental concerns. Overfishing has a major impact on biodiversity loss in our oceans and if current fishing trends continue then fish stocks will be irreparably damaged in the next few decades [12]. An analysis of global fish supply (from wild caught and aquaculture) suggests that there are insufficient fish landings to meet current fish intake recommendations (2–3 portions of fish per week) [13] let alone an entire population consuming a pescatarian diet. The reality of low fish intake is highlighted in the findings that only ~20% of the World’s population consumes the recommended intake of marine derived omega-3 fatty acids [14]. As a result, there has been a significant increase in aquaculture practices [13] and whilst the GHGs associated with fish production are substantially lower than terrestrial meat production [15], some aquaculture practices are associated with significant pollution that may negatively impact the environment and wild fish stocks [16]. This leaves the question; is there an animal-based protein source that is nutritious, associated with low GHG emissions, non-polluting, and does not negatively impact biodiversity when responsibly farmed? The answer may lie in farmed shellfish such as mussels.

### 1.1. The Environmental Benefits of Farmed Mussels

Farmed shellfish is a potentially sustainable alternative to meat, poultry, or fish. There is no requirement for feed and antibiotics for mussel cultivation, and the GHG emissions associated with suspended mussel production are a fraction of that associated with producing terrestrial meat or even farmed salmon, which is considered to have a relatively low GHG output [15] (see Table 1 for comparisons). As a result, the environmental impact of farmed mussel production is considerably lower than all other sources of industrially produced animal meat. By way of comparison for GHG production, eggs produce ~4.5 kg of CO_2_e/kg edible product [17], while mussels only produce 0.6 CO_2_e/kg edible product [15]. Beef, on the other hand, produces ~19.0–36.7 kg of CO_2_e/kg edible product [18].

Furthermore, whilst there is no guarantee that farming mussels is harmless for the environment, if done appropriately then farming mussels can improve the environment [20]. This is partly because mussels grown in suspension require no input in terms of feed and water conditioning chemicals, in addition to the fact that they are filter feeders. Through the regulation of nutrient dynamics, bivalve stocks such as mussels fulfil an important role in the management of coastal water quality [21,22]. Through their filter feeding activity, bivalves mitigate eutrophication and improve water clarity [23]. Globally, it is estimated that cultivated bivalves remove 49,000 tonnes of nitrogen and 6000 tonnes of phosphorous from the oceans, which are the most significant contributors to coastal eutrophication. If this “service” was monetised it is estimated to be worth USD 1.2 billion [24]. There is also significant interest in developing integrated multi-trophic aquaculture (IMTA) systems where species such as salmon are cultivated in close proximity to bivalve species such as mussels [25]. Such IMTA systems would allow for the dual production of mussels and salmon whilst mitigating some of the negative environmental effects of salmon farming [25]. In addition to the environmental value of bivalve aquaculture of species such as mussels, it is estimated that the value of non-food services of bivalve aquaculture is in the region of USD 6 billion [24]. This non-food contribution is from the cultural services provided by bi-valves such as the numerous mussel and seafood festivals held around the world [24]. Due to the potential environmental benefits of increasing the production/consumption of farmed mussels, this review will address some of the potential health benefits of farmed mussels.

### 1.2. The Health Benefits of Mussels

#### 1.2.1. Mussels as a Nutritious Source of Protein

The consumption of bivalves dates to the earliest humans; in Australia, the presence of shell middens that are over 40,000 years old suggests that mussels have likely been consumed here since the first Australians arrived. It is even postulated that our hominid ancestors were able to evolve our characteristic large brains through the exploitation of shellfish in estuarine and coastal environments due in part to the protein and omega-3 content [26]. Whilst catching and consuming sufficient protein and lipid was once a major challenge for our ancestors, a major challenge for our descendants in the 21st century is likely to be producing enough protein to feed the Earth’s growing population [27]. Doing so through terrestrial animal products is likely to be unsustainable. Currently, animal products (meat, poultry, aquaculture, eggs, dairy) account for 37% of the protein and 18% of the calories in our diets [2]. However, the environmental damage of animal products is much greater than plant-based foods; animal products account for 56–58% of food related GHG emissions and occupies 83% of agricultural land use [2]. However, the protein derived from animal products is substantially higher in quality (contain more essential amino acids), digestibility, and bioactivity than protein derived from plant products [17].

Sufficient high-quality sources of protein in the diet are recognised as a critical component to maintain muscle mass, health, and function, and to sustain independence and quality of life into old age [28,29]. Proteins from animal sources also contribute important micro-nutrients (such as bio-available iron and B-12) to the diet that improve overall diet quality [30] and subsequently benefit many other health outcomes including cognitive function [31]. Animal-derived proteins are recognised as the highest quality proteins for optimising muscle health and improving the quality of the diet [17]. Some older adult groups are now encouraged to consume higher than the recommended dietary allowance/intake ((RDA/RDI) 0.8 g/kg/day) for protein to counteract age related muscle loss [32]. Achieving this intake target solely on a plant-based diet would be a challenge due to the low protein/kJ in most plant products when compared to animal products. This also means that to match protein intake from animal sources, significantly larger amounts of plant foods need to be eaten. This may be a challenge for older adults who have higher protein requirements but lower energy requirements (due to immobilisation/inactivity). Furthermore, the higher fibre content of plant foods may further suppress appetite in older adults, who may already have a poor appetite [33]. Thus, achieving higher protein intake targets without consuming excess energy would be much more achievable on a diet that includes animal proteins. Given the aging population of the planet, it is likely that animal proteins will remain an important feature of a healthy diet moving forward, and animal proteins with low GHG emissions may be key to meeting population health and sustainability goals.

Mussels are an excellent source of high-quality protein with an amino acid score of 107 (comparable to the best animal proteins such as eggs which have a score of 100) [34]. Per kJ, mussels have almost as much protein as beef (24 g of protein/600 kJ of lean steak vs. 22 g of protein/600 kJ of mussels) making mussels an ideal substitution for high GHG proteins, particularly red meat. In Table 2, we summarise the nutrient content of key nutrients for a range of protein products including mussels, steak, salmon, and tofu. Mussels outperform other protein sources in many areas, in particular iron and B-12. Salmon outperforms mussels in terms of omega-3 content, but mussels have a much lower GHG contribution than salmon. Whilst mussels have a similar protein content per 100 g of edible portion to tofu, the protein content per kilojoule is better in mussels (22 g of protein per 600 kJ in mussels vs. 15 g of protein per 600 kJ in tofu). This means that mussels can deliver more protein in less energy and with less accompanying fibre than tofu. Whilst mussels are a complete protein with an excellent amino acid score [34] we do not know how well mussel-derived protein stimulates muscle protein synthesis (MPS). The stimulatory potential of dietary proteins on MPS varies greatly [17] and we do not yet know how much mussel protein is required to optimise muscle growth, a key question to answer before firm dietary recommendations based on mussels can be developed.

Environmental arguments for reducing red meat in the diet, whilst compelling, are not the only reason to encourage a reduction in red meat consumption. For instance, to target population heart health in Australia, the National Heart Foundation has suggested that Australians reduce their intake of red meat down from an average of 560 g/week to no more than 350 g/week [35]. To reduce bowel cancer risk, the Cancer Council of Australia has suggested limiting the intake of processed meat [36]. However, a general reduction in meat intake may lead to adverse health effects in some individuals due to a reduction in the intake of high-quality protein and vital nutrients such as iron and B-12. Therefore, a food substitution strategy can help ensure that diet quality is maintained whilst attempting to achieve a healthier dietary pattern. This could be achieved by substituting meat in the diet with farmed mussels.

To demonstrate the impact of such a strategy on nutrient intake we have modelled the impact of replacing 210 g of steak per week with 210 g of blue mussels. Table 3 summarises the impact of this substitution strategy on a range of key nutrients in addition to the impact on GHG emissions of this dietary shift. The edible portion of mussels, at less than 3% fat, is a very lean meat and the modelling demonstrates incorporating 210 g of mussels into the diet in place of steak results in a weekly decrease in energy of 493 kJ (120 kcal). It would also result in a weekly decrease in the intake of total fat (7.3 g) and saturated fat (4 g), whilst simultaneously increasing the intake of long chain omega-3 fatty acids by 1133.9 mg per week. This increase in omega-3 intake equates to 162 mg/day which meets the adequate intake recommendation of 160 mg/day set by the Australian and New Zealand governments (Nutrient Reference Values for Australia and New Zealand. Fats: Total fat and fatty acids. Mussels have a lower protein content per 100 g than lean beef, but the substitution strategy modelled here only results in a small reduction of less than 4 g/day of protein, which is acceptable in light of the overconsumption of protein in typical Western diets. Importantly however, iron intake is increased by 1.1 mg and B12 is increased by 39.9 mg per week. This substitution strategy also results in a reduction in GHGs associated with food of 14.3 kg CO_2_e per week. Thus, finding ecologically valid ways to substitute meat in the diet with farmed mussels could go some way towards meeting health recommendations whilst maintaining diet quality with the additional benefit of helping the environment.

It should be safe for most of the adult population to follow the substitution strategy discussed above, however there are two key micronutrients in relatively high amounts in mussels which certain populations may need to monitor. Iodine content is 267 μg/100 g and whilst the upper limit for iodine intake in adults is 1100 μg/day [37], those with thyroid disorders may need to closely monitor their iodine intake and mussels may therefore be difficult to accommodate in their diet. Secondly, sodium content is 353 mg/100 g thus classifying mussels as “moderate” (<400 mg/100 g) for sodium (Australian Heart Foundation: Heart health education; salt and heart health). As a result, individuals monitoring their sodium intake may not be able to easily accommodate mussels in their diet.

Additional limitations of our suggested strategy are the potential cost and logistical implications of substituting red meat for mussels. There is a large variation in the cost of mussels across and between nations, an analysis of which is out with the scope of this review, but it is possible that some cuts of red meat in some parts of the world may be cheaper than the equivalent weight of mussel meat. Furthermore, there is the logistical challenge of changing an engrained dietary pattern and whilst some mussel products (pre-packaged and pre-cooked) are highly convenient, some products (fresh, raw) are not as convenient to prepare and cook as pre-packaged red meat. These issues would need to be addressed before the above strategy could be widely adopted.

#### 1.2.2. Mussels as a Sustainable Source of Omega-3 Fatty Acids

Due to the health benefits of long chain omega-3 fatty acids, there is an increased demand for omega-3 fatty acid rich products. Recent evidence supports a role for omega-3 in muscle health [29] in addition to cardiovascular, cognitive and bone health [38,39,40,41]. However, only ~20% of the World’s population consumes the recommended 250–500 mg/day of long chain omega-3 [14]. In Australia, the National Heart Foundation recommends that adults aim to consume 250–500 mg of omega-3 fatty acids per day, achieved through consuming two to three servings of fish per week [42]. Recent nationwide dietary analysis suggests that similar to the global numbers, only 20% of Australians are meeting the recommended intake for long chain omega-3 fatty acids [43]. The current recommendations are also problematic as there are currently insufficient fish stocks to sustainably meet the world’s omega-3 fatty acid needs [44]. Recommendations to consume more marine derived omega-3 fatty acids are at odds with sustainability goals due to the environmental impacts of producing farmed fish and the biodiversity impact of fishing. As a result, more sustainable sources of omega-3 fatty acids need to be explored for their potential role in a healthy and sustainable diet.

Farmed mussels have 300–800 mg of omega-3 fatty acids per 100 g of cooked meat [34,45], and 50–60% of the lipid in mussel is phospholipid, meaning that those omega-3 fatty acids are highly bioavailable [46]. Mussels therefore represent a sustainable source of long chain omega-3 fatty acids. Additionally, our research has found that mussel consumption (81–289 g/person/meal depending on study participants’ individual characteristics) as the protein complement of lunch time meals 3 x/week is sufficient to meet the recommended intake of omega-3 and to improve the omega-3 index (a marker of omega-3 intake and heart health) in as little as two weeks [45]. As a result, encouraging the consumption of mussels in place of other proteins could be a viable strategy to sustainably meet omega-3 fatty acid intake recommendations without negatively impacting on the environment. Globally, only ~2 million tonnes of mussels are produced per year [47], which theoretically, for a the global population of ~7 billion, only makes ~300 g of mussels available per person per year. If Oceania is considered seperately, this region produces ~94,000 tonnes of mussels per year [47], meaning that for the Oceania population of ~42 million, ~2.2 kg of mussels is available per person per year. Thus, mussel production would need to be substantially scaled up to provide significant amounts of omega-3 fatty acids to both Oceania and global populations.

#### 1.2.3. Farmed Mussels as a Source of Cholesterol Lowering Phytosterols

Whilst our modelling in Table 3 above suggests that increasing mussel consumption would increase cholesterol intake by 46.3 mg/week, it is possible that mussels might lower cholesterol due to their phytosterol content. Phytosterols are cholesterol like compounds which, similar to cholesterol in mammals, form a key component of plant cell membranes [48]. Thus, phytosterols are found in almost all plant foods but are particularly high in plant-based oils [48]. Due to their similarity to cholesterol, phytosterols inhibit the absorption/reabsorption of dietary cholesterol/bile cholesterol and as a result can lower serum cholesterol levels [48,49,50]. Due to the effect of phytosterols on blood cholesterol, they are often supplemented (as hydrogenated sterols (stanols)) in the diet of individuals seeking to normalise their cholesterol levels [48]. However, some evidence suggests that even non-supplemented phytosterols found naturally in the diet can reduce blood cholesterol [48].

As filter feeders, mussels consume algae and thus accumulate phytosterols in their edible parts [51]. An analysis of two different mussel species; Tasmanian Blue Mussels and New Zealand Green Lipped Mussels, demonstrate that they each contain 20 different phytosterols [52]. Therefore, not only are mussels a good source of heart-healthy omega-3 fatty acids, they also contain cholesterol lowering phytosterols. The next question to ask is: are mussels capable of altering blood lipids? One study suggests that this is a possibility. Childs and colleagues [53] replaced the land-based animal (meat, dairy, eggs) protein in the diet of healthy men with protein from mussels (with the exception of 1 glass of milk per day) for a 7-day period. They found that switching out the land-based animal protein in their diet for an equivalent amount of protein derived from mussels significantly inhibited cholesterol absorption and improved the blood lipid profiles of already normolipidemic men. VLDL (Very Low Density Lipoprotein) triglycerides and VLDL cholesterol were significantly lowered when compared to the control diet and the LDL:HDL (Low Density Lipoprotein:High Density Lipoprotein) ratio was significantly reduced suggesting that mussels, as the sole protein source, can positively impact blood lipid profiles. We cannot be sure from this study if it was a component in the mussels such as the phytosterols or omega-3 fatty acids that led to the changes in blood lipids, or simply the reduction in dietary fat due to the removal of land-based animal products; but the data are compelling, nonetheless. Whilst an extreme example (eating mussels every day for 7 days), the study provides the proof of principle that mussels could form an important part of a heart-healthy diet. Studies employing more ecologically valid strategies of including mussels in the diet, through either supplementation strategies or food replacement protocols, are now needed to address the impact on cardiovascular health markers.

#### 1.2.4. Potential Risks Associated with Mussels

There are numerous food regulatory authorities that operate at global, national, state, and local levels with a view to ensure food safety. All food comes with a degree of risk to human health, but the aim of food regulatory authorities is to minimise that risk and thus ensure that the food we eat is safe. However, despite the regulations in place there are still some real risks to health from certain foods. For instance, processed meat is classified by the International Agency for Research on Cancer as a carcinogen [54] and the Cancer Council of Australia recommends a limit on the intake of processed meats [36]. There are also some food safety concerns with farmed mussels—shellfish allergy likely being the most obvious. A meta-analysis of shellfish allergy suggests that rates of allergy within a population can be anywhere from 0–10.3%, but this depends on the method of diagnosis including self-report questionnaires [55]. When food challenges are used to determine allergy status this rate drops to 0–0.9%. Therefore, whilst less than 1 in 100 people likely have an allergy to mussels, at a global level this represents a substantial number of people who cannot consume them.

As filter feeders, mussels acquire their nutrition by filtering large volumes of the water in which they are suspended. As a result, mussels can accumulate not only nutrients from their environment, but also bacteria, viruses, algal toxins [34,56], micro-plastics [57] and heavy metals [58]. Any one of these factors may present a significant risk to food safety. To combat the risk of bacterial and viral contamination commercial mussel producers cultivate their mussels in waters away from populated/industrial areas. They also put their mussels through depuration [34], a process of placing the mussels in a tank of clean water to allow them to empty their gastrointestinal tracts and thus remove grit, bacteria and viruses [56]. Depuration and water monitoring for toxic algal blooms substantially reduces (but does not eliminate) the risk of food poisoning from commercial shellfish [34,56].

Depuration can also reduce the contamination from microplastics by almost 30% [59]. However, the risk posed by microplastics in the food chain is still very much unknown [57], and they seem to be endemic in the food supply chain [60]. For reference, bottled water has been reported to have a microplastic load of 2600–6300 microplastics/litre [60], while mussels have been reported to have a microplastic load of 0.36–0.47 microplastics/g wet weight (equivalent to 360–470 microplastics/kg wet weight of mussels) [57]. Without attempting to trivialise the risk of microplastics in the food chain, a meal of mussels will likely contain less microplastics than a glass of bottled water.

The other general concern with seafood is the risk of toxic trace metal contamination (cadmium, arsenic, lead and mercury are of main concern). Seafood does accumulate trace metals, particularly the further up the trophic chain the animal sits. Since mussels are herbivores, they tend to have less trace metal contamination than top carnivores such as sword fish. However, in a study on the accumulation of heavy metals in the blood of men fed 1 kg of fish and mussels per week for 26 weeks, it was found that lead accumulated above a tolerable exposure concentration [61]. That said, the authors do point out that the background intake for lead was “already disturbingly high” and the increase in lead from the intervention whilst significant was small. Additionally, none of the other trace metals (cadmium, arsenic, mercury) increased to a level that would cause concern [61]. In another study on mussels collected in the Adriatic Sea the authors calculated the provisional tolerable weekly intake (PTWI) for mussels [58]. The PTWI estimates how much of a substance can be consumed on a weekly basis over the course of a lifetime without incurring negative health effects. For mussels from the Adriatic Sea the amount was limited by the cadmium content and equated to 640–1200 g of mussel meat per week [58]. Furthermore the authors calculated the risk of trace metal toxicity from consuming 125 g of mussels per week and determined that none of the metals investigated (zinc, iron, copper, nickel, cadmium, lead, arsenic and mercury) posed a risk to human health [58].

Whilst the trace metal content of mussels will vary considerably by location [62] and season [63], cultivated mussels are typically grown in areas of low pollution and in Australia, commercially cultured mussels are sampled and analysed prior to going to market [63]. The Australia and New Zealand Food Standards (FSANZ) Code limits cadmium and lead levels in mollusks to 2 mg/kg wet weight [64]. Therefore, based on the approach of Jovic et al. [58] it is likely safe to eat 200 g of Australian mussels per week without exceeding the PTWI for cadmium. However, this is based on the FSANZ upper limit of 2 mg/kg of wet weight of mussels and the cadmium levels are likely much lower from commercial mussels harvested in Australia. This assumption is based on data from mussels that are often used as sentinel organisms to monitor the water for trace metal contamination. In one such study the authors transplanted mussels from a commercial mussel cultivator in South Australia. They sampled the transplanted mussels prior to deployment to establish a baseline trace metal contamination. The baseline cadmium concentration was reported to be ~1.25 µg/g dry weight, which calculated on the dry weight to wet weight conversions used in Shen et al. [63], is equivalent to 0.53 mg/kg wet weight [65]. Therefore, guided by data on cadmium intakes from these commercial mussels it would be safe to consume close to 800 g of these mussels per week. Whilst the conclusions of Jovic et al. [58] apply to mussels landed from the Adriatic Sea, it is likely that similar conclusions can be drawn for commercially cultivated mussels in Australia.

The risk of eating shellfish to those suffering from shellfish allergy cannot possibly be outweighed by the nutritional benefits of consuming mussels. However, those who can enjoy mussels can likely do so safely in the knowledge that any risks of eating commercially cultivated mussels are likely outweighed by the substantial nutritional benefits. Indeed, Mozaffarian et al. [66] reviewed the available evidence on the risks from consuming seafood (including shellfish) covering everything from dioxins, polychlorinated biphenyls, to heavy metal exposure, and concluded the benefits to health of seafood consumption outweighed the risks. Similarly, Vengopal et al. [34] reviewed the risk-benefit evidence specifically on shellfish, covering pathogen contamination to heavy metal contamination, and again concluded the nutritional benefits of eating shellfish outweigh the risks.

## 2. Conclusions

Sustainably feeding our planet’s growing population in a way that meets changing nutritional needs throughout the lifespan is going to be a complex challenge for the 21st century. This challenge will require multiple integrated solutions [27], and a key component is likely to involve reducing our reliance on land animal protein [4]. However, a vegan diet, whilst a laudable goal, is unlikely to be a solution due to the population’s varying food preferences. Furthermore, a vegan diet is unlikely to support everyone’s nutritional needs. Thus, we suggest that farmed mussels may be part of the solution. They are a highly nutritious alternative to land animal protein, but they have a very low environmental footprint when compared to terrestrial animal protein. Substituting some of the land animal protein in our diets with mussels could have the dual benefit of improving heart health (particularly if substituted for red meat) and reducing the GHG emissions associated with our diet. Based on several analyses of the risks of seafood/shellfish [34,58,66], replacing 210 g of red meat with 210 g of commercially cultivated mussels is highly likely to be safe in the long term. Of course, farmed mussel production is unlikely ever to be at a level that would feed 10 billion people, and not everyone can enjoy mussels because of allergies and food preferences, but those who can enjoy mussels could consider substituting some of the land animal protein in their diet with farmed mussels. In so doing, we will reduce our environmental footprint and potentially improve our nutritional intake and overall health.

## Figures and Tables

**Table 1:** A comparison of protein content and GHG emissions in kgCO_2_e per kg of edible product. GHG numbers are extracted from [15,17,18,19] and protein data are extracted from Australian Food Nutrient data base.

Protein Product	Protein Content per 100 g of Cooked and Edible Product	kg of GHGs per kg of Edible Product
Beef	27.0	19.0–36.7
Lamb	27.5	23.0–36.0
Pork	30.6	6.4–8.6
Poultry	29.8	3.0–6.5
Salmon	29.2	4.2–5.4
Eggs	14.1	4.5
Tofu	16.4	0.1
Blue Mussels	16.0	0.6

**Table 2:** Summary of nutrient content and GHG emissions per 100 g of edible portion of mussels, steak, salmon and tofu. Nutrient data are extracted from the Australian Food Nutrient data base and the GHG data are extracted from [15,18,19].

Per 100 g Edible Portion	Blue Mussels, Cooked, No Added Fat	Steak, Fully Trimmed, Cooked, No Added Fat	Salmon, Cooked, No Added Fat	Tofu (Soy Bean Curd), Cooked, No Added Fat
Energy, without fibre (kJ)	438.00	673.00	1202.00	649.00
Protein (g)	16.00	27.00	29.20	16.40
Dietary fibre (g)	0.00	0.00	0.00	4.80
Riboflavin (B2) (mg)	0.07	0.19	0.14	0.09
Niacin (B3) (mg)	0.73	5.14	4.48	0.65
Niacin derived equivalents (mg)	3.77	8.85	11.14	3.83
Dietary folate equivalents (µg)	23.00	0.00	0.00	39.00
Vitamin B6 (mg)	0.08	0.12	0.75	0.12
Vitamin B12 (µg)	20.00	1.00	2.50	0.00
Vitamin C (mg)	5.00	1.00	0.00	0.00
Alpha-tocopherol (mg)	1.00	0.90	5.00	0.00
Vitamin E (mg)	1.05	0.86	4.97	0.00
Calcium (Ca) (mg)	173.00	6.00	10.00	438.00
Iodine (I) (µg)	267.80	1.10	9.80	3.80
Iron (Fe) (mg)	2.97	2.45	1.45	3.97
Magnesium (Mg) (mg)	76.00	27.00	34.00	107.00
Phosphorus (P) (mg)	122.00	246.00	361.00	329.00
Potassium (K) (mg)	131.00	381.00	428.00	178.00
Selenium (Se) (µg)	96.00	10.40	30.30	6.80
Sodium (Na) (mg)	353.00	55.00	57.00	55.00
Zinc (Zn) (mg)	3.12	4.66	0.42	2.33
Cholesterol (mg)	94.00	72.00	90.00	0.00
Total saturated fat (g)	0.37	2.26	3.98	1.32
Total monounsaturated fat (g)	0.20	2.46	7.40	2.24
Total polyunsaturated fat (g)	0.79	0.44	5.66	5.58
Linoleic acid (g)	0.04	0.20	1.56	5.00
Alpha-linolenic acid (g)	0.06	0.06	0.63	0.58
C20:5w3 EPA (mg)	191.33	28.38	1268.27	0.00
C22:5w3 DPA (mg)	21.47	40.09	606.05	0.00
C22:6w3 DHA (mg)	400.87	5.25	1184.51	0.00
Total long chain omega 3 fatty acids (mg)	613.67	73.72	3058.83	0.00
Total trans fatty acids (mg)	46.20	246.33	284.61	0.00
GHG emission (kg CO_2_)	0.06	1.90–3.67	0.42–0.54	0.01

**Table 3:** Modelling the impact of replacing 210 g of steak with 210 g of blue mussels (edible portion). The nutrition data were extracted (February 2021) from the Australian Food and Nutrient data base (Food Standards Australia New Zealand (FSANZ) (2014). AUSNUT 2011–2013—Australian Food and Nutrient Database 2011–2013. Canberra). We modelled the red meat on lean steak with fat trimmed and no added fat for cooking and we modelled the blue mussels on mussels cooked with no added fat. The GHG data are extracted from [15,18] and we used the middle of the range of GHGs (24.899 kg CO_2_e/kg of meat) reported for beef based on the range 19–36.7 kg CO_2_e/kg of beef.

Per 100 g	Steak 560 g/Week (Current Red Meat Intake in Australia)	Steak 350 g/Week (AHF Recommended Intake) + 210 g/Week Blue Mussels (Substitution)	Delta Difference
Energy, without dietary fibre (kJ)	3768.80	3275.30	−493.50
Protein (g)	151.20	128.10	−23.10
Total fat (g)	32.48	25.13	−7.30
Vitamin A retinol equivalents (µg)	16.80	130.20	113.40
Thiamin (B1) (mg)	0.20	0.12	−0.08
Riboflavin (B2) (mg)	1.07	0.81	−0.26
Niacin (B3) (mg)	28.78	19.52	−9.30
Niacin derived equivalents (mg)	49.56	38.89	−10.70
Vitamin B6 (mg)	0.67	0.59	−0.08
Vitamin B12 (µg)	5.60	45.50	39.90
Vitamin C (mg)	5.60	14.00	8.40
Alpha-tocopherol (mg)	5.04	5.25	0.20
Vitamin E (mg)	4.82	5.22	0.40
Calcium (Ca) (mg)	33.60	384.30	350.70
Iron (Fe) (mg)	13.72	14.81	1.10
Magnesium (Mg) (mg)	151.20	254.10	102.90
Selenium (Se) (µg)	58.24	238.00	179.80
Zinc (Zn) (mg)	26.10	22.86	−3.24
Cholesterol (mg)	403.20	449.40	46.20
Total saturated fat (g)	12.66	8.68	−4.00
Total monounsaturated fat (g)	13.78	9.03	−4.75
Total polyunsaturated fat (g)	2.46	3.20	0.74
Linoleic acid (g)	1.12	0.78	−0.30
Alpha-linolenic acid (g)	0.34	0.34	0.00
C20:5w3 EPA (mg)	158.93	501.12	342.19
C22:5w3 DPA (mg)	224.50	185.40	−39.10
C22:6w3 DHA (mg)	29.40	860.20	830.80
Total long chain omega 3 fatty acids (mg)	412.84	1546.72	1133.88
Total trans fatty acids (mg)	1379.45	959.18	−420.27
GHG emission (kg CO_2_)	39.20	24.90	−14.3

## References

[1] Czyżewski B., Kryszak Ł. (2018). Impact of different models of agriculture on greenhouse gases (GHG) emissions: A sectoral approach. Outlook Agric..

[2] Poore J., Nemecek T. (2018). Reducing food’s environmental impacts through producers and consumers. Science.

[3] Herrero M., Havlík P., Valin H., Notenbaert A., Rufino M.C., Thornton P.K., Blümmel M., Weiss F., Grace D., Obersteiner M. (2013). Biomass use, production, feed efficiencies, and greenhouse gas emissions from global livestock systems. Proc. Natl. Acad. Sci. USA.

[4] Aleksandrowicz L., Green R., Joy E.J., Smith P., Haines A. (2016). The impacts of dietary change on greenhouse gas emissions, land use, water use, and health: A systematic review. PLoS ONE.

[5] Berners-Lee M., Hoolohan C., Cammack H., Hewitt C. (2012). The relative greenhouse gas impacts of realistic dietary choices. Energy Policy.

[6] Willett W., Rockström J., Loken B., Springmann M., Lang T., Vermeulen S., Garnett T., Tilman D., DeClerck F., Wood A. (2019). Food in the Anthropocene: The EAT–Lancet Commission on healthy diets from sustainable food systems. Lancet.

[7] Dinu M., Abbate R., Gensini G.F., Casini A., Sofi F. (2017). Vegetarian, vegan diets and multiple health outcomes: A systematic review with meta-analysis of observational studies. Crit. Rev. Food Sci. Nutr..

[8] Craig W.J. (2009). Health effects of vegan diets. Am. J. Clin. Nutr..

[9] Tong T.Y.N., Appleby P.N., Armstrong M.E.G., Fensom G.K., Knuppel A., Papier K., Perez-Cornago A., Travis R.C., Key T.J. (2020). Vegetarian and vegan diets and risks of total and site-specific fractures: Results from the prospective EPIC-Oxford study. BMC Med..

[10] Norman K., Klaus S. (2020). Veganism, aging and longevity: New insight into old concepts. Curr. Opin. Clin. Nutr. Metab. Care.

[11] Burdge G.C., Tan S.-Y., Henry C.J. (2017). Long-chain n-3 PUFA in vegetarian women: A metabolic perspective. J. Nutr. Sci..

[12] Worm B., Barbier E.B., Beaumont N., Duffy J.E., Folke C., Halpern B.S., Jackson J.B., Lotze H.K., Micheli F., Palumbi S.R. (2006). Impacts of biodiversity loss on ocean ecosystem services. Science.

[13] Thurstan R.H., Roberts C.M. (2014). The past and future of fish consumption: Can supplies meet healthy eating recommendations?. Mar. Pollut. Bull..

[14] Micha R., Khatibzadeh S., Shi P., Fahimi S., Lim S., Andrews K.G., Engell R.E., Powles J., Ezzati M., Mozaffarian D. (2014). Global, regional, and national consumption levels of dietary fats and oils in 1990 and 2010: A systematic analysis including 266 country-specific nutrition surveys. BMJ.

[15] Fry J.M. (2012). Carbon Footprint of Scottish Suspended Mussels and Intertidal Oysters.

[16] Bergland H., Burlakov E., Pedersen P.A., Wyller J. (2020). Aquaculture, pollution and fishery-dynamics of marine industrial interactions. Ecol. Complex..

[17] Gorissen S.H., Witard O.C. (2018). Characterising the muscle anabolic potential of dairy, meat and plant-based protein sources in older adults. Proc. Nutr. Soc..

[18] Suplicy F.M. (2020). A review of the multiple benefits of mussel farming. Rev. Aquac..

[19] Mejia A., Harwatt H., Jaceldo-Siegl K., Sranacharoenpong K., Soret S., Sabaté J. (2018). Greenhouse gas emissions generated by tofu production: A case study. J. Hunger Environ. Nutr..

[20] Gallardi D. (2014). Effects of bivalve aquaculture on the environment and their possible mitigation: A review. Fish. Aquac. J..

[21] Jansen H.M., Strand Ø., Van Broekhoven W., Strohmeier T., Verdegem M.C., Smaal A.C. (2019). Feedbacks from filter feeders: Review on the role of mussels in cycling and storage of nutrients in oligo-meso-and eutrophic cultivation areas. Goods and Services of Marine Bivalves.

[22] Petersen J.K., Holmer M., Termansen M., Hasler B. (2019). Nutrient extraction through bivalves. Goods and Services of Marine Bivalves.

[23] Cranford P.J. (2019). Magnitude and extent of water clarification services provided by bivalve suspension feeding. Goods and Services of Marine Bivalves.

[24] van der Schatte Olivier A., Jones L., Vay L.L., Christie M., Wilson J., Malham S.K. (2020). A global review of the ecosystem services provided by bivalve aquaculture. Rev. Aquac..

[25] Stenton-Dozey J.M., Heath P., Ren J.S., Zamora L.N. (2020). New Zealand aquaculture industry: Research, opportunities and constraints for integrative multitrophic farming. N. Z. J. Mar. Freshw. Res..

[26] Crawford M.A. (2002). Cerebral Evolution. Nutr. Health.

[27] Smil V. (2001). Feeding the World: A Challenge for the Twenty-First Century.

[28] McLeod M., Breen L., Hamilton D.L., Philp A. (2016). Live strong and prosper: The importance of skeletal muscle strength for healthy ageing. Biogerontology.

[29] Witard O.C., McGlory C., Hamilton D.L., Phillips S.M. (2016). Growing older with health and vitality: A nexus of physical activity, exercise and nutrition. Biogerontology.

[30] Phillips S.M., Fulgoni III V.L., Heaney R.P., Nicklas T.A., Slavin J.L., Weaver C.M. (2015). Commonly consumed protein foods contribute to nutrient intake, diet quality, and nutrient adequacy. Am. J. Clin. Nutr..

[31] Wengreen H.J., Neilson C., Munger R., Corcoran C. (2009). Diet quality is associated with better cognitive test performance among aging men and women. J. Nutr..

[32] Bauer J., Biolo G., Cederholm T., Cesari M., Cruz-Jentoft A.J., Morley J.E., Phillips S., Sieber C., Stehle P., Teta D. (2013). Evidence-based recommendations for optimal dietary protein intake in older people: A position paper from the PROT-AGE Study Group. J. Am. Med. Dir. Assoc..

[33] Kaur D., Rasane P., Singh J., Kaur S., Kumar V., Mahato D.K., Dey A., Dhawan K., Kumar S. (2019). Nutritional Interventions for Elderly and Considerations for the Development of Geriatric Foods. Curr. Aging Sci..

[34] Venugopal V., Gopakumar K. (2017). Shellfish: Nutritive Value, Health Benefits, and Consumer Safety. Compr. Rev. Food Sci. Food Saf..

[35] Foundation H. (2019). Meat and Heart Healthy Eating: Dietary Position Statement.

[36] Australia C.C. (2019). Position Statement-Meat and Cancer Prevention.

[37] Trumbo P., Yates A.A., Schlicker S., Poos M. (2001). Dietary Reference Intakes: Vitamin A, Vitamin K, Arsenic, Boron, Chromium, Copper, Iodine, Iron, Manganese, Molybdenum, Nickel, Silicon, Vanadium, and Zinc. J. Acad. Nutr. Diet..

[38] Calder P.C. (2018). Very long-chain n-3 fatty acids and human health: Fact, fiction and the future. Proc. Nutr. Soc..

[39] Ferguson J.J., Veysey M., Lucock M., Niblett S., King K., MacDonald-Wicks L., Garg M.L. (2016). Association between omega-3 index and blood lipids in older Australians. J. Nutr. Biochem..

[40] Harris W.S., Von Schacky C. (2004). The Omega-3 Index: A new risk factor for death from coronary heart disease?. Prev. Med..

[41] Sadeghi O., Djafarian K., Ghorabi S., Khodadost M., Nasiri M., Shab-Bidar S. (2019). Dietary intake of fish, n-3 polyunsaturated fatty acids and risk of hip fracture: A systematic review and meta-analysis on observational studies. Crit. Rev. Food Sci. Nutr..

[42] Foundation N.H. (2008). Position Statement, Fish., Fish. Oils, n-3 Polyunsaturated Fatty Acids and Cardiovascular Health.

[43] Meyer B. (2016). Australians are not meeting the recommended intakes for omega-3 long chain polyunsaturated fatty acids: Results of an analysis from the 2011–2012 national nutrition and physical activity survey. Nutrients.

[44] Brunner E.J., Jones P.J., Friel S., Bartley M. (2009). Fish, human health and marine ecosystem health: Policies in collision. Int. J. Epidemiol..

[45] Carboni S., Kaur G., Pryce A., McKee K., Desbois A.P., Dick J.R., Galloway S.D.R., Hamilton D.L. (2019). Mussel Consumption as a “Food First” Approach to Improve Omega-3 Status. Nutrients.

[46] Ahmmed M.K., Ahmmed F., Tian H., Carne A., Bekhit A.E.-D. (2020). Marine omega-3 (n-3) phospholipids: A comprehensive review of their properties, sources, bioavailability, and relation to brain health. Compr. Rev. Food Sci. Food Saf..

[47] Wijsman J.W.M., Troost K., Fang J., Roncarati A., Smaal A.C., Ferreira J.G., Grant J., Petersen J.K., Strand Ø. (2019). Global Production of Marine Bivalves. Trends and Challenges. Goods and Services of Marine Bivalves.

[48] Ostlund R.E. (2002). Phytosterols in human nutrition. Annu. Rev. Nutr..

[49] Eilat-Adar S., Sinai T., Yosefy C., Henkin Y. (2013). Nutritional recommendations for cardiovascular disease prevention. Nutrients.

[50] Ntanios F.Y., Jones P.J. (1999). Dietary sitostanol reciprocally influences cholesterol absorption and biosynthesis in hamsters and rabbits. Atherosclerosis.

[51] Hailat I.A., Parrish C.C., Helleur R.J. (2016). Sterol Composition of Blue Mussels Fed Algae and Effluent Diets from Finfish Culture. J. Shellfish Res..

[52] Murphy K.J., Mooney B.D., Mann N.J., Nichols P.D., Sinclair A.J. (2002). Lipid, FA, and sterol composition of New Zealand green lipped mussel (Perna canaliculus) and Tasmanian blue mussel (Mytilus edulis). Lipids.

[53] Childs M.T., Dorsett C.S., King I.B., Ostrander J.G., Yamanaka W.K. (1990). Effects of shellfish consumption on lipoproteins in normolipidemic men. Am. J. Clin. Nutr..

[54] Domingo J.L., Nadal M. (2017). Carcinogenicity of consumption of red meat and processed meat: A review of scientific news since the IARC decision. Food Chem. Toxicol..

[55] Moonesinghe H., Mackenzie H., Venter C., Kilburn S., Turner P., Weir K., Dean T. (2016). Prevalence of fish and shellfish allergy: A systematic review. Ann. Allergy Asthma Immunol..

[56] Jackson K., Ogburn D. (1999). Review of Depuration and Its Role in Shellfish Quality Assurance.

[57] Van Cauwenberghe L., Janssen C.R. (2014). Microplastics in bivalves cultured for human consumption. Environ. Pollut..

[58] Jović M., Onjia A., Stanković S. (2012). Toxic metal health risk by mussel consumption. Environ. Chem. Lett..

[59] Birnstiel S., Soares-Gomes A., da Gama B.A.P. (2019). Depuration reduces microplastic content in wild and farmed mussels. Mar. Pollut. Bull..

[60] van Raamsdonk L.W.D., van der Zande M., Koelmans A.A., Hoogenboom R.L.A.P., Peters R.J.B., Groot M.J., Peijnenburg A.A.C.M., Weesepoel Y.J.A. (2020). Current Insights into Monitoring, Bioaccumulation, and Potential Health Effects of Microplastics Present in the Food Chain. Foods.

[61] Outzen M., Tjønneland A., Larsen E.H., Hansen M., Andersen K.K., Christensen J., Overvad K., Olsen A. (2015). Effect of increased intake of fish and mussels on exposure to toxic trace elements in a healthy, middle-aged population. Food Addit. Contam. Part A.

[62] Richardson B.J., Garnham J.S., Fabris J.G. (1994). Trace metal concentrations in mussels (Mytilus edulis planulatus L.) transplanted into Southern Australian waters. Mar. Pollut. Bull..

[63] Shen H., Kibria G., Wu R.S.S., Morrison P., Nugegoda D. (2020). Spatial and temporal variations of trace metal body burdens of live mussels Mytilus galloprovincialis and field validation of the Artificial Mussels in Australian inshore marine environment. Chemosphere.

[64] Authority A.-A.N.Z.F. (2003). Australia New Zealand Food Standards Code-Standard 1.4. 1-Contaminants and Natural Toxicants.

[65] Gaylard S., Thomas S., Nelson M. (2011). An assessment of the current status of bioavailable metal contamination across South Australia using translocated mussels Mytilus Galloprovincalis. Trans. R. Soc. S. Aust..

[66] Mozaffarian D., Rimm E.B. (2006). Fish intake, contaminants, and human health: Evaluating the risks and the benefits. JAMA.

